# Utilizing geospatial artificial intelligence to map cancer disparities across health regions

**DOI:** 10.1038/s41598-024-57604-y

**Published:** 2024-04-02

**Authors:** Ahmed Fadiel, Kenneth D. Eichenbaum, Mohammad Abbasi, Nita K. Lee, Kunle Odunsi

**Affiliations:** 1 Computational Oncology Unit, University of Chicago Medicine Comprehensive Cancer Center, 900 E 57th St, KCBD Bldg., Chicago, IL 60637 USA; 2 Department of Anesthesiology, Oakland University William Beaumont School of Medicine, 3601 W. 13 Mile Rd, Royal Oak, MI 48073 USA; 3 University of Chicago Medicine Comprehensive Cancer Center, 5841 South Maryland Avenue, MC1140, Chicago, IL 60637 USA; 4 Department of Obstetrics and Gynecology, University of Chicago, Chicago, IL 60637, USA

**Keywords:** Cancer, Computational biology and bioinformatics, Diseases, Health care

## Abstract

We have developed an innovative tool, the Intelligent Catchment Analysis Tool (iCAT), designed to identify and address healthcare disparities across specific regions. Powered by Artificial Intelligence and Machine Learning, our tool employs a robust Geographic Information System (GIS) to map healthcare outcomes and disease disparities. *iCAT* allows users to query publicly available data sources, health system data, and treatment data, offering insights into gaps and disparities in diagnosis and treatment paradigms. This project aims to promote best practices to bridge the gap in healthcare access, resources, education, and economic opportunities. The project aims to engage local and regional stakeholders in data collection and evaluation, including patients, providers, and organizations. Their active involvement helps refine the platform and guides targeted interventions for more effective outcomes. In this paper, we present two sample illustrations demonstrating how *iCAT* identifies healthcare disparities and analyzes the impact of social and environmental variables on outcomes. Over time, this platform can help communities make decisions to optimize resource allocation.

## Introduction

There is a significant gap in the ability of researchers to understand how disease progression and treatment vary across communities. We have designed a platform for a searchable health application across various regions that embeds artificial intelligence and machine learning to bridge this gap. Specifically, our *iCAT* (Intelligent Catchment Analysis Tool) platform analyzes data from local areas with disease disparities to reduce gaps in education and treatment. Given the diverse population groups and our available resources, we elected to populate this AI-enhanced geographically oriented software platform with data from the catchment area around the University of Chicago Medicine Comprehensive Cancer Center. We describe (1) the motivation, background and our specific initial focus around cancer for the application design, (2) methods of software, statistics and AI features, (3) results and discussion of example use cases of how the application permits users to answer health related disparity questions and (4) limitations, summary thoughts and future research direction.

### Driving principles of the platform

Health professionals require improved platforms to communicate with the public and develop well-designed methods to benefit and protect public health^1^. The core tenets of these platforms draw from The National Academy of Sciences, Engineering, and Medicine’s 2019 report on *Integrating Social Care into the Delivery of Healthcare,* which characterizes several activities that aid in better integrating social needs into healthcare^2,3^. These activities include promoting awareness, handling social barriers, assisting patients in obtaining educational resources, aligning healthcare systems with needs, and establishing advocacy partnerships. Recently, the ability to apply Big Data to study remote sensing and mapping technologies has dramatically improved^4^ and enhanced our understanding of geographic heterogeneity across different regions^5^.

### Motivation for applying AI to dynamic geographic health data

The complex and dynamic nature of healthcare data poses a challenge for conventional analytical methods to extract actionable insights. AI's innate adaptability offers transformative potential in healthcare, facilitating real-time analysis for swift identification of emerging health trends and timely interventions. This paradigm shift promises to revolutionize healthcare decision-making and outcomes, showcasing the power of AI/ML to solve this challenge.

GIS technology utilizes spatial data and maps to analyze and visualize patterns and trends within a geographic context^6^. High-level analysis can help map the environmental elements associated with disease disparities and offer evidence-based support for logical diagnosis and treatment plans^7^. Studies have demonstrated that GIS can integrate various data types, including demographic, environmental, and healthcare information, to understand the complex factors contributing to disease disparities^8^. Over time, as users query increasingly complex datasets, the data may contain nonlinear dynamics that require advanced algorithmic approaches to organize and efficiently optimize data analysis^9^. It may also be of wide interest for users to employ intelligent neuro-supervised networks (INSNs) to study variations across different regional locations^10^. The accuracy and analytical speed of analysis of large geographic datasets can benefit from enhanced fractional stochastic gradient descent (EFSGD) approaches that employ matrix factorization to improve prediction accuracy^11^.

Integrating AI with GIS enables us to pinpoint areas with high disease prevalence, study environmental and social factors contributing to cancer disparities, and assist in developing evidence-based prevention and treatment strategies. By incorporating the data from localized catchment areas with advanced treatment modalities and comparing it to areas with poor outcomes, GIS can help researchers and health systems understand the root causes of health disparities and develop interventions to reduce them.

### New application design

We designed the *iCAT* application with intelligence to allow users to visualize geospatial health data on a map and perform ML/AI analysis with valid embedded models. By leveraging demographic, environmental, and healthcare access data, users can perform sophisticated univariate and multivariate AI models to identify patterns and trends in disease incidence rates. This application can identify barriers to accessing screening and treatment, analyze survey and interview data, and inform policy and interventions to address healthcare disparities.

### Mapping health disparities by disease type using specialized cancers as a model

Cancer disparities refer to the unequal distribution and burden of cancer among different population groups, often based on race, ethnicity, socioeconomic status, and geographic location. To build a comprehensive health disparity analysis tool that includes all cancer types, we focused initially on gynecologic cancers, where known outcome disparities have been reported in the literature^12^. Gynecologic cancers, including cervical, ovarian, uterine, and vulvar cancer, have been identified as a subgroup of interest for GIS studies. In 2018, there were an estimated 570,000 new cases of cervical cancer and 311,000 deaths worldwide, with nearly 90% of patients being from low and middle-income countries^13,14^. The advent of human papillomavirus (HPV) vaccines for high-risk subtypes of cervical cancer and early interventions in at-risk populations provide effective treatment options when identified early^15^.

By analyzing data from the GIS platform, researchers, community advocates, health systems, and legislative bodies can address the root causes of health disparities and improve disease outcomes. It is important to note that statistical knowledge is necessary to evaluate the significance of relationships and correlations between factors. While these tests are robust, guidance from a statistician may be helpful to verify model assumptions, including confounding factors or data transformations that may impact correlation size.

## Methods

### Defining a geographic catchment area

We defined our initial boundary geography as the University of Chicago Medicine Comprehensive Cancer Center (UCCCC) catchment area, a five-county area around the University of Chicago Medical Center on the south side of Chicago. This area includes 83% of UCCCC cancer patients and 62% of clinical trial patients. The included counties are Cook County, IL, the broader Chicago metropolitan area; DuPage County, IL; Will County, IL; Lake County, IL; and Lake County, IN. These counties have the highest population density in IL and IN and an estimated combined population of approximately 8 million. The population of the total catchment area has a racial and ethnic breakdown of residents as 49% non-Hispanic white, 22% Hispanic, 19% Black, and 8% Asian. This catchment area has a high rate of poverty and social deprivation, known barriers to cancer screening, timely and quality cancer care, and limited resources for cancer survivorship.

### Data collection and availability

Deidentified health, demographic, socio-economic, and environmental data were obtained from publicly available relevant sources, such as the Chicago Health Atlas^16^ and the Chicago Data Portal^17^. Spatial cancer mortality data related to the catchment area were collected and matched with demographic, socio-economic, health, and spatial data and are available for use in the *iCAT* application.

### Software

*iCAT* is built using R statistical software package (v 4.2.1) (2022–6-23) available at: https://cran.r-project.org/bin/windows/Rtools/rtools42/rtools.html^18^ and the R shiny package (v 1.7.4) available at: https://www.rdocumentation.org/packages/shiny/versions/1.7.4^19^. The leaflet r package (v 2.1.2)^20^ is an R implementation of the leaflet JavaScript library used for plotting GIS data on a map. The caret package (v 6.0–94) and its dependencies in R perform the bulk of the AI/ML analysis and is available at https://www.rdocumentation.org/packages/caret/versions/6.0-94.

### iCAT user interface

The platform uses RShiny to build a user-friendly interface optimized for simplicity. A sidebar with specific tabs for each task allows the user to navigate around and efficiently perform the required analysis. Moreover, a guide tab is included to acquaint the user with the different functionalities of the tool, and an in-app example dataset is provided to help the user study cancer outcomes within the catchment area. The application incorporates tabs that report the results of various analyses, including mapping visual distributions, correlation, and multivariate. This enables users to study the results of multiple analysis methods simultaneously and draw informative conclusions.

### GIS health data visualization

Visualization of the data on a map of the boundary area is performed using the leaflet package. This package allows the visualization of different layers on the same map to study multiple variables simultaneously. It works efficiently across all desktop and mobile operating systems, with an interactive experience and the capability to adjust the map's scale to focus on a catchment area of interest. The plotting data render numeric variables using a gradient coloring scale, implying a continuous spectrum across the values. In contrast, categorical variables are presented with each category as entirely distinct.

### Univariate analysis and statistical tests

*iCAT* uses univariate analysis to aid in understanding the effect of a single variable on a health outcome (i.e., cancer mortality). It identifies the root causes of health disparities and improves cancer outcomes. Examples of this are vast and can range from studying the relationship between smoking or socio-economic status on lung cancer mortality to the relation between routine check-ups and cervical cancer mortality. *iCAT* provides statistical measures and tests that can be readily used to test the significance of univariate relations. For example, the size of the Pearson correlation between smoking and lung cancer can be calculated and returned as a measure of the relation between the two variables. A t-test can be performed to study the significance of this correlation measure.

Univariate analysis can be performed by selecting the primary and secondary variables and choosing Pearson's or Spearman’s correlation as the analysis method to measure the correlation between the two variables. Appropriate statistical tests can be performed to assess the significance of the correlation. The results of the correlation analysis are presented using the raw data after using *iCAT*’s statistical AI to perform data transformations and remove outliers in the variables. *iCAT*’s statistical AI provides the user with an overall evaluation of the model, including checks for confounding factors, which helps thoroughly assess results without advanced statistical experience.

### Multivariate analysis using machine learning

The application uses multivariate analysis to assess the effect of multiple variables on the outcome variable simultaneously to provide a better understanding of the contribution of various social and environmental variables to health disparity outcomes. ML systems can accept many dimensions and explain essential features instead of analyzing them manually one at a time. *iCAT* provides access to various linear and non-linear machine learning algorithms (such as linear regression, logistic regression, GBMs, Neural Networks, and Lasso Regression) based on the problem and reports the significant features. It ranks them based on their impact on the response of interest. Multivariate analysis can be performed by selecting the primary and secondary variables in the analysis tab and selecting the required analysis method.

Multiple machine learning algorithms in *iCAT* allow the user to select the analysis that fits the data and health question of interest. Linear regression models are used to study the linear relationship of various social and environmental variables on continuous health disparity outcomes (i.e. cancer mortality rate)^21^, while logistic regression provides the user with the ability to study important factors affecting the presence or absence of health disparity outcomes^22^. GBMs and Neural Networks are powerful AI models that can study non-linear relationships between various social and environmental variables and health disparity outcomes^23,24^. Lasso regression models are also available to allow the user to study the effect of many correlated variables on the health outcome without worrying about confounding factors^25^. The statistical AI in the app’s back end allows the user to provide the data and lets the app select the most appropriate multivariate method to analyze the data.

### iCAT statistical AI

Intelligent statistical and data analysis relies on AI to test assumptions and troubleshoot any issues related to the data evaluation. Techniques used in this process include but are not limited to data cleaning and preprocessing (i.e., removing outliers), data engineering and transformation (i.e., transforming to normal distribution), knowledge filtering (i.e., eliminating confounding factors), recommendation systems (i.e., recommending important health disparity factors in cancer outcomes). These methods are utilized in the statistical AI present in *iCAT* to give the user an easy way to gain confidence in the statistical and machine learning models and analysis results presented.

Several unique features built into *iCAT* provide intelligent data analysis, including data transformation, data processing, confounding checks, and disparity recommendations, and are described below.

### Data transformation

**Figure 1:**
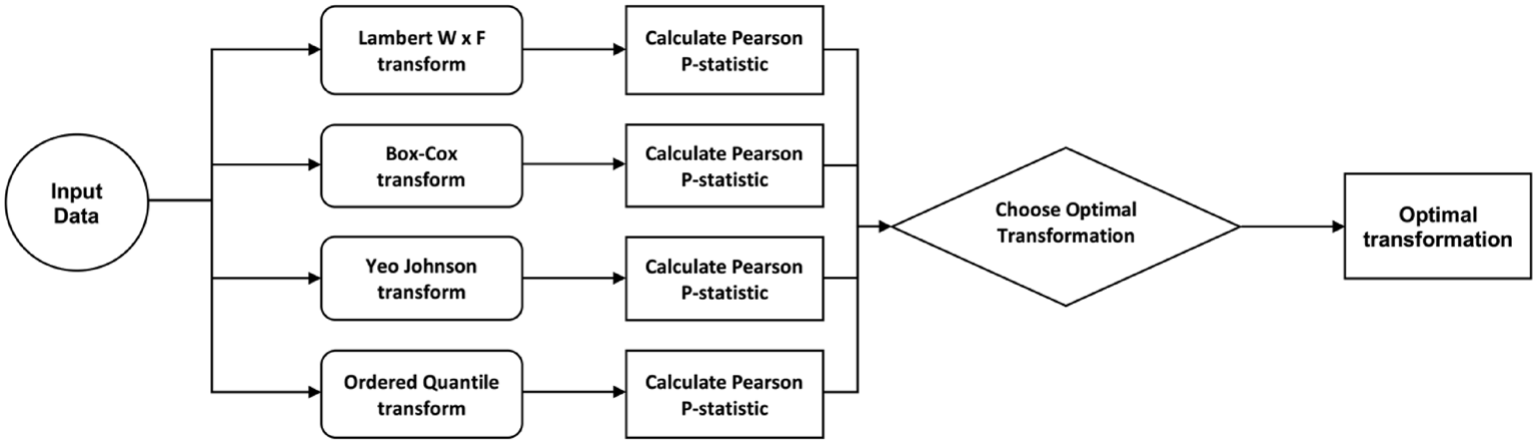
The process of choosing the best-fit normality transform for the input data.

The data selected for analysis is assessed to ensure correct assumptions of the analysis method and proper data transformations are applied when necessary. This is enabled using the tool’s underlying statistical AI and the bestNormalize R package. Appropriate regular transformations are initially applied to the input data from well-known available transformations such as Lambert W x F transform, Box-Cox transform, Yeo-Johnson transforms, and Ordered Quantile. The best transformation is then selected by calculating the Pearson P statistic divided by its degrees of freedom for each transformation. The transformation that allows the data to best-fit normality is selected and applied to the data (Fig. 1).

### Data cleaning and outlier removal

The normal transformed data are cleaned by removing potential outliers. The outliers are selected using the interquartile method. This method first calculates the interquartile range (IQR) as the distance between the 1^st^ and 3^rd^ quartiles (Q1-Q3). It then assumes any value outside the range [Q1- 1.5 × IQR, Q3 + 1.5 × IQR] is an outlier. Outliers are removed from the dataset to avoid potential interference with the analysis results.

### Confounding factors

The presence of potential confounding factors that can affect the result of the statistical models is assessed, and the results before and after accounting for confounding factors are presented to the user. This is possible due to the app's focus on the set catchment area and the comprehensive demographic and metadata available to assess confounding.

The presence of confounding factors is assessed using linear models in the following way: Initially, a single variable linear model is fit using the primary variable of interest (i.e., Cancer mortality) as the response variable and the second variable in the univariate analysis as the covariate in the model. *iCAT* then includes internal metadata columns as potential confounders to the model. Subsequently, the percent change in the coefficient of the second variable in the first and second linear models is calculated. The generally accepted practice finds that a percentage change exceeding 10% can point to the potential presence of confounders or other variables that explain the variance in the primary variable of interest^26^. *iCAT* reports the result of this analysis in addition to the correlation matrix between the variables of interest and the potential confounders for the user to view. It also provides the potential confounders and calculates the correlation after accounting for confounding variables.

### Recommendations for health disparity factors in cancer outcomes

*iCAT* can provide recommendations on important demographic, socio-economic, and environmental factors on a given cancer outcome based on a statistical reference to similar factors in the selected area of interest. The desired primary outcome can be programmatically estimated using the appropriate ML multivariate analysis method to identify relevant factors in determining the cancer outcome. The app can 'learn' using the data in the area of interest. This can help scientists understand hidden health disparity factors and recommend interventions promptly that can lead to better prevention of cancer-related mortality*.*

## Results and discussion

The *iCAT* geo-analyzer is a publicly available online interactive tool at: https://cancercatchmentareas.godaddysites.com/

The *iCAT* geo-analyzer enables users to quantify disease burden in cancer over multiple years. Users can filter the results by year, age group, and a minimum number of diagnoses. Users can also study the disparity in cancer burden in the catchment area using different statistical and ML analysis methods. This paper presents two use examples of the application to investigate cancer burden in the University of Chicago Comprehensive Cancer Center service catchment area.

## Example 1: Evaluating disparities in cervical cancer mortality rate in Cook County, IL

**Figure 2:**
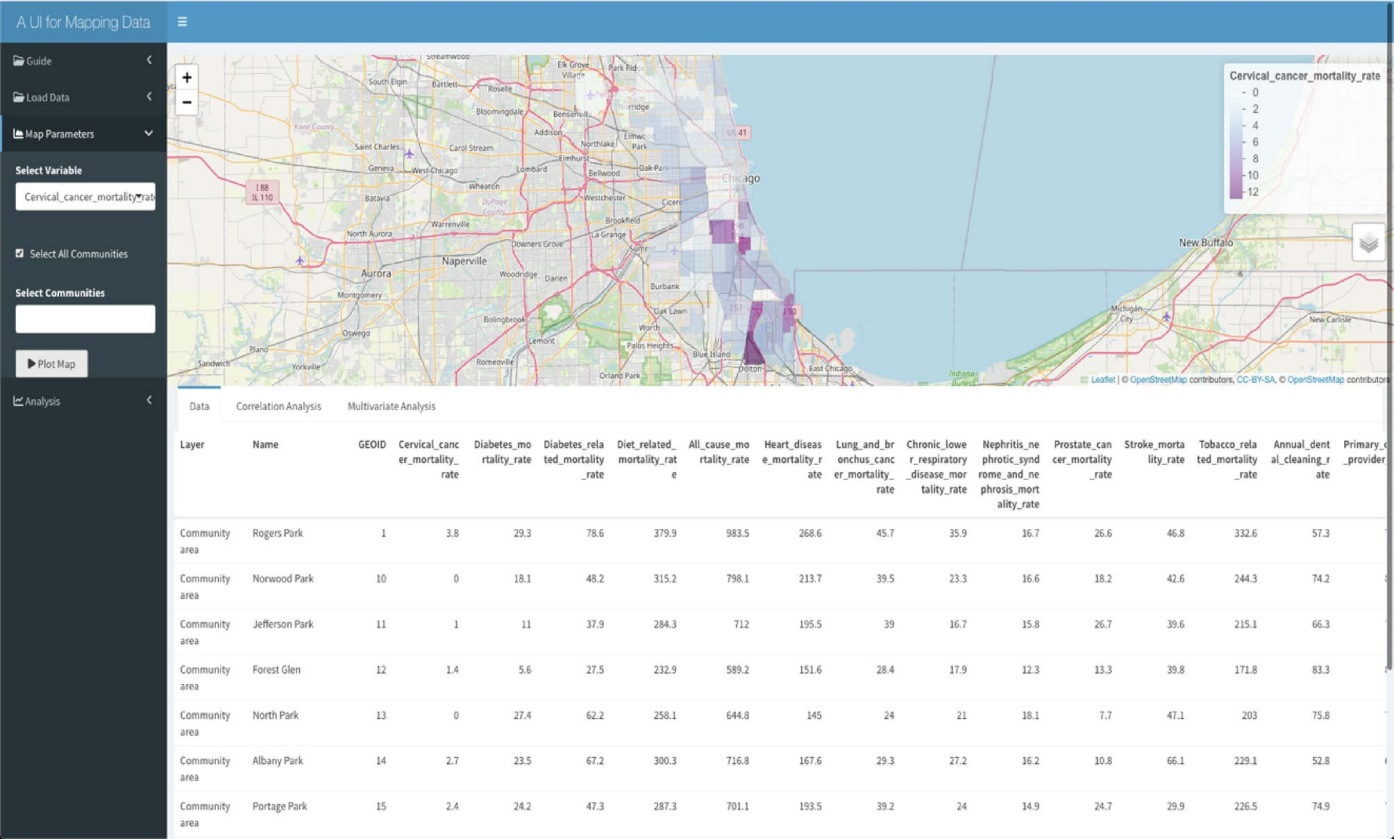
The overlay of cervical cancer mortality rate on the map of the UCCC-CA. Mapping: The map was made using the leaflet r package version leaflet_2.1.2 and the OpenStreetMap tile. leaflet: URL: https://CRAN.R-project.org/package=leaflet; GPL license for the leaflet R package https://cran.r-project.org/web/licenses/GPL-3; OpenStreetMap: Copyright and license for open access: https://www.openstreetmap.org/copyright#:~:text=OpenStreetMap%C2%AE%20is%20open%20data,credit%20OpenStreetMap%20and%20its%20contributors.

Using the *iCAT* application to study this question, the distribution of cervical cancer mortality rates in different communities in Cook County, IL, can be seen in Fig. 2. The communities are colored by the value of the selected variable, with darker purple showing higher values of the selected variable. Using correlation analysis in *iCAT*, we can test the correlation between cervical cancer mortality and poverty rates. This is done by choosing the cervical cancer mortality rate as the primary and the poverty rate as the secondary variable and assigning the Pearson Correlation method from the drop-down menu in the Analysis tab.

Table 1 demonstrates two different correlation analysis results. First, Pearson’s correlation coefficient was calculated using the raw data. Second, Pearson’s correlation coefficient was calculated after using the underlying statistical AI to clean and transform data to the appropriate format expected by the analysis method. After applying statistical intelligence, a highly significant correlation coefficient of 0.48 is reported. In the next step, *iCAT* checks for potential confounders using in-app demographic and socio-economic data from the catchment area (Table 2).

**Table 1:** Pearson’s correlation analysis results between cervical cancer mortality and the poverty rate.

Method	Label	Correlation	95% CI	t	*p*-value
Pearson’s product-moment correlation	Raw Data	0.568	[0.395 0.703]	5.982	0
Pearson’s product-moment correlation	Using Statistical Intelligence	0.481	[0.288 0.636]	4.747	0

**Table 2:** Pearson’s correlation analysis results after removing potential confounders.

Method	Label	Correlation	95% CI	t	*p*-value
Pearson’s product-moment correlation	Confounding Removed	0.049	[−0.177 0.27]	0.427	0.6704

**Figure 3:**
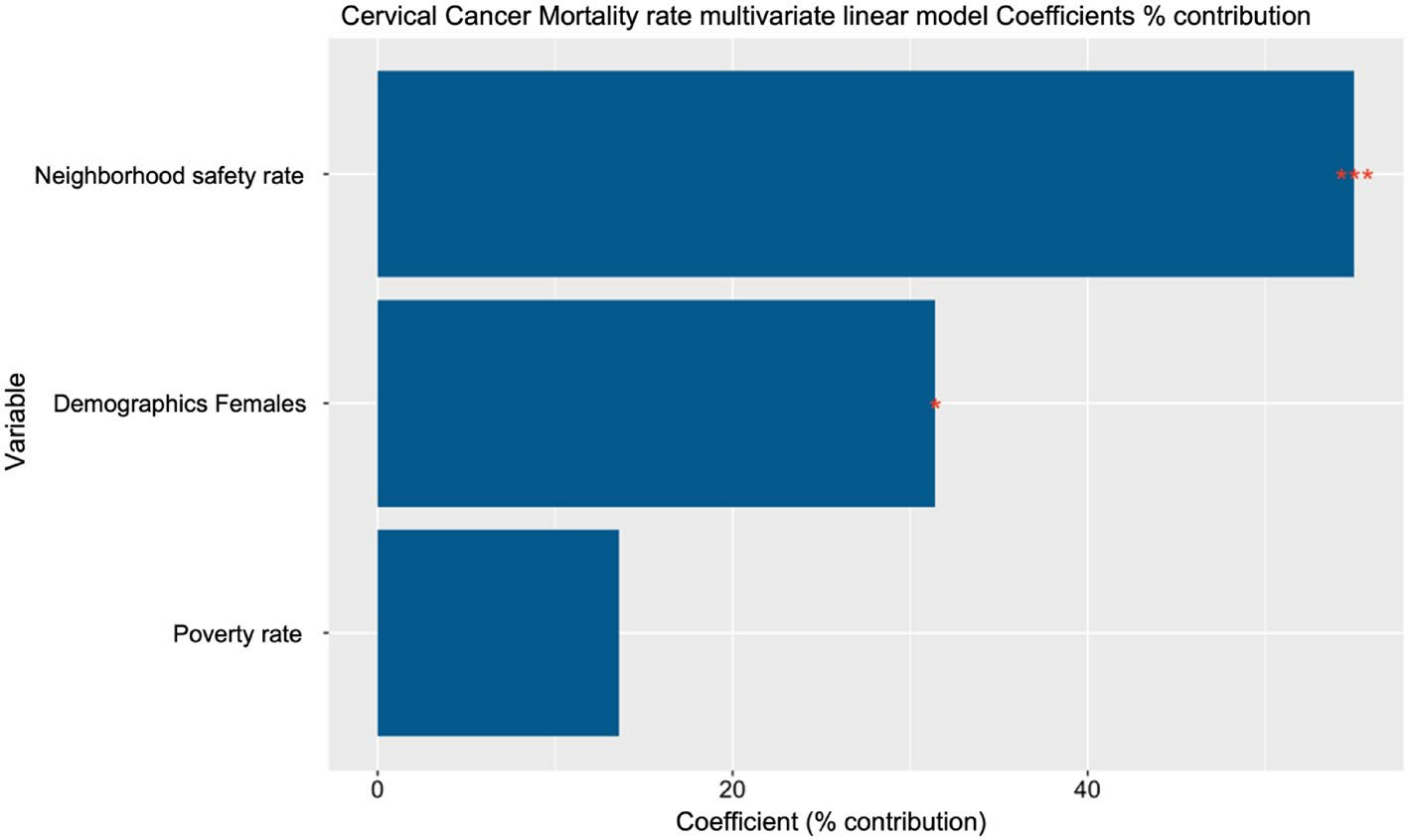
Multivariate analysis between cervical cancer mortality rate, poverty rate, and potential confounders.

After removing the effect of potential confounders from the primary variable, we identify no significant correlation between cervical cancer mortality and poverty rate. Close observation of the correlation matrix shows that the community's female demographic and neighborhood safety rate are potential confounders of the analysis, so we need to account for these dependencies when studying cervical cancer. *iCAT* allows multivariate analysis to study the effect of multiple variables on the cervical cancer mortality rate simultaneously. This feature can study the effect of poverty rate on cervical cancer mortality while controlling for confounders. The multivariate analysis selects the cervical cancer mortality rate as the primary variable and the poverty rate, female demographics, and neighborhood safety rate as the secondary variables. For this analysis, using a simple linear regression model, the results of the multivariate analysis show that when controlling for the female demographic, the neighborhood safety rate is a significant contributor to explaining the cervical cancer mortality rate. In contrast, the poverty rate shows no significant contribution (Table 3, Fig. 3). This exciting result points out the neighborhood safety rate as a contributor to the cervical cancer mortality rate rather than the poverty rate.

**Table 3:** Multivariate analysis results between cervical cancer mortality rate, poverty rate, and potential confounders.

Feature	Coefficients	Coeff (%) contribution	t.value	*P*.value	R^2^ adjusted
Poverty_rate	0.112	13.6	0.801	0.426	0.432
Neighborhood_safety_rate	−0.450	55.0	−3.550	0.001	0.432
Demographics_Females	0.257	31.4	2.557	0.013	0.432

## Example 2: Studying disparities in overall cancer mortality rate in Cook County, IL

**Figure 4:**
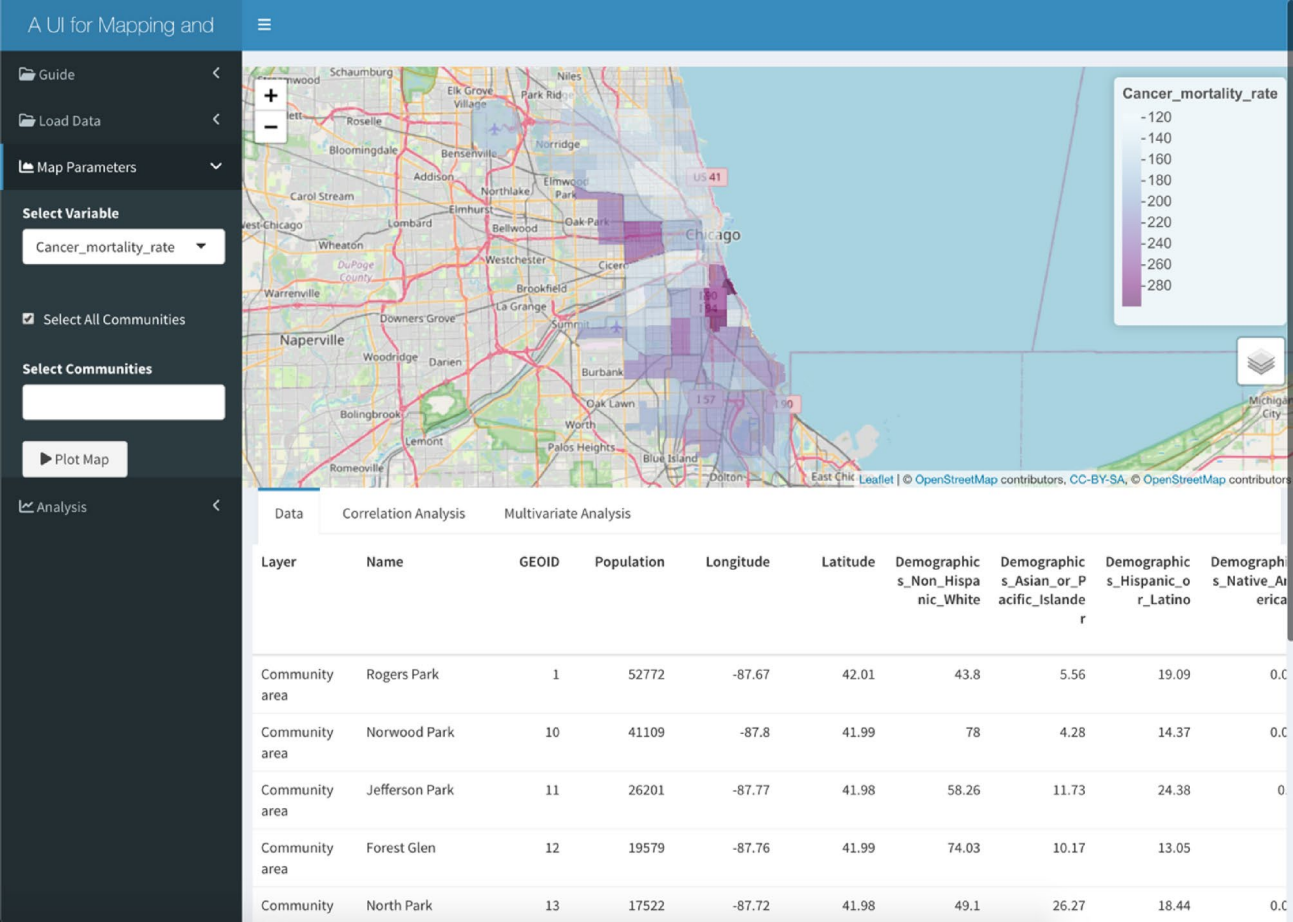
The overlay of cancer mortality rate on the map of the University of Chicago Medicine Comprehensive Cancer Center service area. Mapping: The map was made using the leaflet r package version leaflet_2.1.2 and the OpenStreetMap tile. leaflet: URL: https://CRAN.R-project.org/package=leaflet; GPL license for the leaflet R package https://cran.r-project.org/web/licenses/GPL-3; OpenStreetMap: Copyright and license for open access: https://www.openstreetmap.org/copyright#:~:text=OpenStreetMap%C2%AE%20is%20open%20data,credit%20OpenStreetMap%20and%20its%20contributors.

The distribution of cancer mortality rates in different communities in Cook County, IL, can be visualized in Fig. 4. Using multivariate analysis in *iCAT*, we can test the relation between cancer mortality and multiple demographics, socio-economic variables, and other variables. This is done by choosing the cancer mortality rate as the primary factor, and demographic, socio-economic, and other factors as the secondary variables and assigning the linear regression method from the drop-down menu in the Analysis tab.

**Figure 5:**
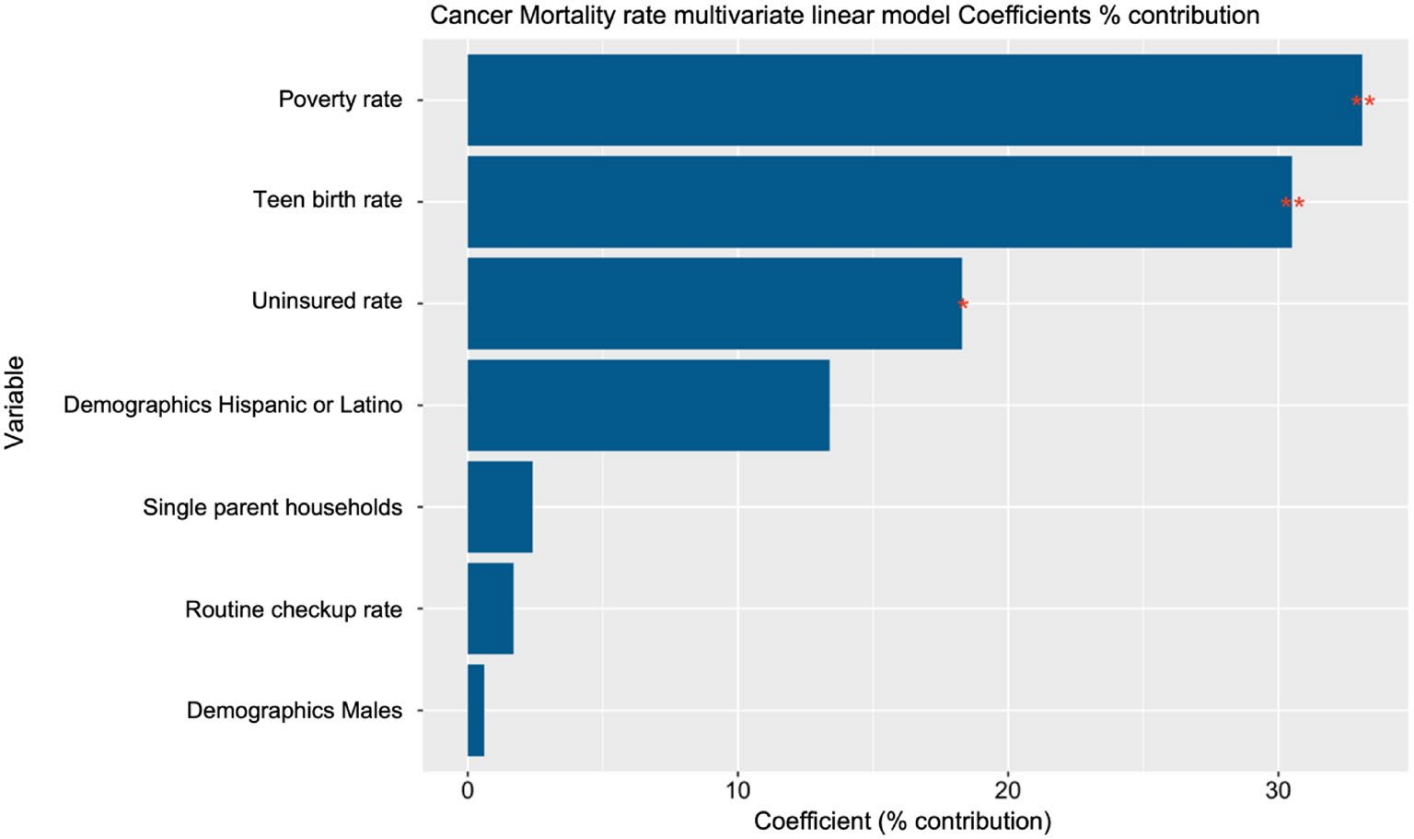
The results of using a linear regression model to study the effect of multiple demographic, socio-economic, and other factors on overall cancer mortality in the University of Chicago Medicine Comprehensive Cancer Center service area. Feature Importance scores are the percent contribution to the linear regression model.

The importance of the secondary variables in explaining the overall cancer mortality rate in the designated area can be seen in Fig. 5 and Table 4. In this case, three factors significantly contribute to the overall cancer mortality rate: poverty and teen birth rates show a positive relationship, and the uninsured rate negatively correlates with overall cancer mortality.

**Table 4:** The results of using a linear regression model to study the effect of multiple demographic, socio-economic, and other factors on overall cancer mortality in the University of Chicago Medicine Comprehensive Cancer Center service area.

feature	Coefficients	Coeff (%) contribution	t.value	*P*.value	R^2^ adjusted
Poverty_rate	0.432	3.31	2.831	0.006	0.596
Teen_birth_rate	0.398	30.5	2.817	0.006	0.596
Uninsured_rate	−0.238	18.3	−2.016	0.048	0.596
Demographics_Hispanic_or_Latino	−0.175	13.4	−1.423	0.159	0.596
Routine_checkup_rate	−0.023	1.7	−0.257	0.798	0.596
Single_parent_households	0.031	2.4	0.178	0.859	0.596
Demographics_Males	−0.007	0.6	−0.061	0.952	0.596

The example illustrations show the importance of *iCAT* in studying disease outcomes in health disparity populations. In a similar situation, many available GIS applications may have reported the result of a positive correlation between cervical cancer mortality and poverty rates. By accounting for potential confounders, the statistical AI in *iCAT* allows us to make more reliable inferences and avoid costly mistakes due to incorrect analysis results. Using the innovative GIS in this tool, we expect to identify patterns and trends in health disparity results within the catchment area. We also hope to identify potential social and environmental factors contributing to these disparities.

This GIS project can utilize data on demographic characteristics, lifestyle behaviors, and environmental exposures to identify patterns and trends in disease incidence and outcomes. An AI-GIS analysis may reduce the disease burden by revealing higher cervical cancer rates in specific neighborhoods by discovery of environmental exposures or lifestyle behaviors. This can help community leaders implement interventions such as increasing access to screening and education on risk reduction strategies.

## Limitations

Presently, we have populated only a narrow data set into the model, which restricts the dynamic capabilities of the application along the machine learning overlays. A comprehensive dataset will further improve the performance of the tool’s underlying statistical AI, which, in turn, improves the accuracy of the data analysis results. Over time, incidence data, demographic information, environmental exposure data, healthcare access, and quality of care will be expanded and can provide a higher-powered dataset to harvest. Another limitation is that we still need to integrate logic to verify the data sources are reliable and up-to-date.

## Conclusion

AI-GIS platforms offer a powerful tool to pinpoint drivers of health disparities. We demonstrated two examples of the impact of social and environmental variables on cervical cancer mortality and overall cancer mortality rates. This type of analysis can serve as a template for leveraging big data to provide useful conclusions about regional disparities. Ultimately, enhanced AI algorithmic strategies and output analysis can drive tactical resource allocation to reduce disparities over time. We demonstrated how the AI-GIS platform can answer user queries about specific health disparity information and uncover the finding that neighborhood safety rate contributes to the cervical cancer mortality rate rather than the poverty rate.

## Future directions

In addition to analyzing data on one disease within one region, larger-scale data analysis can encompass many diseases and multiple communities and/or capture specific population characteristics across multiple regions. For example, a GIS analysis may reveal higher rates of ovarian cancer in some areas of the country, potentially linked to environmental exposures or lifestyle behaviors. Based on this information, policymakers or community leaders can implement interventions such as increasing access to screening and education on risk reduction strategies at a national level.

## Data Availability

The datasets generated and/or analyzed during the current study are available in the Dryad repository, https://datadryad.org/stash/share/KpwSya3YeR1cG32z3Bs_FTv3SSyRf7j4S6cAXdT3qDI

## References

[1] Carroll LN, Au AP, Detwiler LT, Fu TC, Painter IS, Abernethy NF (2014). Visualization and analytics tools for infectious disease epidemiology: A systematic review. J. Biomed. Inform..

[2] Ngongo WM, Peterson J, Lipiszko D, Gard LA, Wright KM, Parzuchowski AS, Ravenna PA, Cooper AJ, Persell SD, O'Brien MJ, Goel MS (2023). Examining how social risk factors are integrated into clinical settings using existing data: A scoping review. Ann. Fam. Med..

[3] National Academies of Sciences, Engineering, and Medicine . Integrating Social Care Into the Delivery of Health Care: Moving Upstream to Improve the Nation’s Health. The National Academies Press (2019). 10.17226/25467 31940159

[4] Prasad A, Gray CB, Ross A, Kano M (2016). Metrics in urban health: Current developments and future prospects. Annu. Rev. Public Health..

[5] Soares RR (2022). The evolving field of Big Data: understanding geographic information systems analysis and its transformative potential in ophthalmic research. Curr. Opin. Ophthalmol..

[6] Cromley EK (2003). GIS and disease. Annu. Rev. Public Health..

[7] Clair K, Bristow RE (2021). Looking at cancer health disparities in gynecologic oncology in 2020. Curr. Opin. Obstet. Gynecol..

[8] Fletcher-Lartey SM, Caprarelli G (2016). Application of GIS technology in public health: successes and challenges. Parasitology..

[9] Chaudhary NI, Khan ZA, Kiani AK, Raja MAZ, Chaudhary II, Pinto CM (2022). Design of auxiliary model based normalized fractional gradient algorithm for nonlinear output-error systems. Chaos Solitons Fractals..

[10] Mukhtar R, Chang C-Y, Raja MAZ, Chaudhary NI (2023). Design of intelligent neuro-supervised networks for brain electrical activity rhythms of Parkinson’s disease model. Biomimetics.

[11] Khan ZA, Chaudhary NI, Khan TA, Farooq U, Pinto CMA, Raja MAZ (2023). Enhanced fractional prediction scheme for effective matrix factorization in chaotic feedback recommender systems. Chaos Soliton Fract.

[12] Collins Y, Holcomb K, Chapman-Davis E, Khabele D, Farley JH (2014). Gynecologic cancer disparities: a report from the Health Disparities Taskforce of the Society of Gynecologic Oncology. Gynecol. Oncol..

[13] Arbyn M, Weiderpass E, Bruni L, de Sanjosé S, Saraiya M, Ferlay J, Bray F (2020). Estimates of incidence and mortality of cervical cancer in 2018: a worldwide analysis. Lancet Glob. Health..

[14] Cohen PA, Jhingran A, Oaknin A, Denny L (2019). Cervical cancer. Lancet..

[15] Kamolratanakul S, Pitisuttithum P (2021). Human papillomavirus vaccine efficacy and effectiveness against cancer. Vaccines (Basel)..

[16] Chicago Health Atlas. https://chicagohealthatlas.org. January 9 (2024).

[17] Chicago Data Portal, https://data.cityofchicago.org/browse?tags=gis

[18] R Core Team, R: A Language and Environment for Statistical Computing, R Foundation for Statistical Computing, Vienna, Austria (2021). https://www.R-project.org/

[19] Chang, W., Cheng, J., Allaire, J., Sievert, C., Schloerke, B., Xie, Y., Allen, J., McPherson, J., Dipert, A., Borges, B. *shiny: Web Application Framework for R*. R package version 1.8.0.9000 (2023). https://github.com/rstudio/shiny, https://shiny.posit.co/.

[20] Cheng, J., Karambelkar, B., Xie, Y. *leaflet: Create Interactive Web Maps with the JavaScript 'Leaflet' Library*. R package version 2.1.1 (2022). https://rstudio.github.io/leaflet/.

[21] Lai, T. L_etc, Robbins, H., & Zi Wei, C. Strong consistency of least squares estimates in multiple regression II. *J. Multivar. Anal.* **9.3** 343-361 (1979). 10.1073/pnas.75.7.3034 PMC392707 16592540

[22] Cox DR (1958). The regression analysis of binary sequences. J. R. Stat. Soc.: Ser. B (Methodological).

[23] Friedman, J. H. Greedy function approximation: A gradient boosting machine. *Ann. Stat.* 1189–1232 (2001).

[24] McCulloch WS, Pitts W (1943). A logical calculus of the ideas immanent in nervous activity. Bull Math Biophys..

[25] Tibshirani, R. Regression shrinkage and selection via the lasso. *J. R. Stat. Soc. Ser. B (Methodological)* (1996): 267–288.

[26] Budtz-Jørgensen E, Keiding N, Grandjean P, Weihe P (2007). Confounder selection in environmental epidemiology: Assessment of health effects of prenatal mercury exposure. Ann. Epidemiol..

